# Combining RSSI and Accelerometer Features for Room-Level Localization

**DOI:** 10.3390/s21082723

**Published:** 2021-04-13

**Authors:** Athina Tsanousa, Vasileios-Rafail Xefteris, Georgios Meditskos, Stefanos Vrochidis, Ioannis Kompatsiaris

**Affiliations:** Centre for Research and Technology Hellas, Information Technologies Institute, 6th Km Charilaou-Thermi, 57001 Thessaloniki, Greece; vxefteris@iti.gr (V.-R.X.); gmeditsk@iti.gr (G.M.); stefanos@iti.gr (S.V.); ikom@iti.gr (I.K.)

**Keywords:** room-level localization, RSSI, inertial sensors, fusion

## Abstract

The continuing advancements in technology have resulted in an explosion in the use of interconnected devices and sensors. Internet-of-Things (IoT) systems are used to provide remote solutions in different domains, like healthcare and security. A common service offered by IoT systems is the estimation of a person’s position in indoor spaces, which is quite often achieved with the exploitation of the Received Signal Strength Indication (RSSI). Localization tasks with the goal to locate the room are actually classification problems. Motivated by a current project, where there is the need to locate a missing child in crowded spaces, we intend to test the added value of using an accelerometer along with RSSI for room-level localization and assess the performance of ensemble learning methods. We present here the results of this preliminary approach of the early and late fusion of RSSI and accelerometer features in room-level localization. We further test the performance of the feature extraction from RSSI values. The classification algorithms and the fusion methods used to predict the room were evaluated using different protocols applied to a public dataset. The experimental results revealed better performance of the RSSI extracted features, while the accelerometer’s individual performance was poor and subsequently affected the fusion results.

## 1. Introduction

Internet-of-Things systems consist of multiple connected devices. The interconnected devices interact and produce large amounts of exploitable data that are used to improve lives in various aspects [1]. One of the common services found in IoT systems is localization or Location-Based Services (LBS). Localization services can be quite useful in digital health applications [2], market [3], and security domain applications [4]. Depending on the space where it takes place, localization can be categorized as indoor and outdoor. Indoor spaces are considered more difficult for such tasks due to the existence of obstacles, like walls and objects that affect the accurate position estimation of a target. In outdoor spaces, we usually find systems that exploit the global positioning system (GPS) while in indoor localization the most popular technology is the use of radio transmissions like WiFi and Bluetooth beacons [5].

Indoor localization approaches can be further categorized as Range-Based solutions that use techniques like RSSI, Time-of-Arrival (TOA), and Range-Free solutions that are based on the distance of the sensor nodes [6]. RSSI measures the distance between a transmitter and a receiver [7]. Its availability in many devices makes it an easy choice for localization tasks. One of the disadvantages is that it is quite subject to noise and a filtering method is needed to adjust the RSSI values [7]. Usually, Kalman filtering techniques are applied to raw RSSI signals to eliminate noise. In the current application, instead of applying the usual Kalman or particle filtering, we adopted the feature extraction from RSSI values that was proposed in [8]. Feature extraction is usually found in the wearable sensors’ analysis. The current work intends to combine RSSI with an accelerometer, to achieve room-level localization, thus the features were extracted from both RSSI and accelerometer values.

The choice of methodologies depends on whether we need to estimate the accurate position of a target, e.g., their coordinates, or the room they are in, which is usually referred to as room-level localization. When estimating the distance of a target to a reference point, there is usually a combination of a technique that translates the RSSI to distance, like the path loss model, and methods like triangulation or trilateration that estimate the real coordinates of the target using three reference nodes [7]. The room-level localization requires classification algorithms.

To improve room-level localization, wearable sensors that record movement can be used in combination with the RSSI. The accelerometer that is used in the current work, is a sensor very commonly exploited in IoT systems. Accelerometers are embedded in almost all smartphones and smartwatches. They can be found in affordable devices and they are widely used in the recognition of activities, actions, gestures, and critical situations, such as fall detection. Accelerometers are the most effective sensors in activity recognition tasks. Another use of this sensor is as a step counter [9] and when used in combination with RSSI or similar technologies it can improve the localization.

In the present work, we extract features from the RSSI readings as well and apply a variety of fusion methods and classifiers to investigate the performance of RSSI and accelerometers in room-level localization both in combination and separately. More specifically, we present the results of the individual analysis of each modality and the fusion of two modalities at the early level as well as at the results’ level, using two evaluation protocols. We assess the performance of early and late fusion methods borrowed from the machine learning field, instead of the usual Kalman filtering approaches used in such problems. To the best of our knowledge, the early and late fusion of RSSI and accelerometer values for room-level localization is quite limited [10], in terms of applications, as well as datasets. The publicly available BOX dataset [11], was chosen to apply the evaluation protocols since it contains RSSI and accelerometer readings, as well as room labels. The dataset consists of separate sets for training and testing and is intended for room-level localization, which means that the true labels include only the room where a user is, not the exact distance relative to the RSSI receiver. Ten users participated in the collection of the data. The users wore an accelerometer device that recorded the activity they conducted in each room. One specific activity was conducted in each room so that the rooms and activities fully coincide and the accelerometer recordings can be used for room recognition. We applied feature extraction from RSSI and accelerometer values to eliminate the noise and afterward combined the features of the two sources to predict the room. We used well-known fusion methods of classifiers to combine the heterogeneous recordings of RSSI and an accelerometer.

Our work has been motivated by the research challenges involved in public safety contexts, where the aim is to timely respond to emergencies, such as natural disasters, health-related incidents, vandalism, and missing children in overcrowded places. The latter is one of the targeted use cases in the DESMOS (http://desmos-project.gr/, accessed on 8 April 2021) project, developing intelligent sensor-based technologies to estimate the location of children in one of the most crowded Christmas thematic parks in Greece that takes place in December in Trikala (Mills of the Elves). Children wear a smart bracelet with Bluetooth Low Energy (BLE) technology which is wirelessly connected with the parent’s phone. The challenge is to accurately estimate the location of the child, taking into account all the available modalities (RSSI and accelerometer values). We could summarize the contributions of this work in the following:

- Testing the efficiency of accelerometer measurements in room-level localization task, which is actually a classification problem.
- Examining the performance of feature extraction from RSSI readings, based on the features suggested in [8].
- Assessing the performance of RSSI and accelerometer data together, in room-level localization, by combining them in an early level or in the results level. To achieve this, we applied several ensemble learning methods, which are not usually implemented in such tasks, but they are very common in wearable sensors’ analysis for problems like activity recognition or fall detection.
- We manipulated the initial dataset [11] in two different ways, which we later refer to as evaluation protocols, to apply the aforementioned framework. We finally compare the individual performance of RSSI and accelerometer features, with the performance of the concatenated features (early fusion) as well as the performance of the late fusion algorithms.

The rest of the paper is organized as follows: Section 2 revises the related work. In Section 3 we describe the methods used. Section 4 describes the application and the experimental results. Section 5 discusses the results of the project and in the final section, the conclusions are presented.

## 2. Related Work

Indoor localization has been assessed with various methods. From these methods, RSSI is the simplest to be implemented in applications. RADAR [12], a radio-frequency based system for locating and tracking users inside buildings, is one of the first indoor positioning systems using a WiFi-based RSSI fingerprinting method. The system has an average of three meters error on the coordinate of two dimensions. The authors in [13] also use WiFi-based fingerprinting to perform accurate indoor localization. Their system aims to improve simple fingerprinting by using not only the position information but also the orientation. This method achieved a resolution of 1.8 m.

Various techniques have been developed to improve the performance of RSSI-based indoor localization. In [14] a hybrid model based on heat maps generated from WiFi RSSI signals was proposed. The model takes advantage from both a convolutional neural network (CNN) and a long short-term memory network (LSTM) and outperforms other typical WiFi RSSI signal-based localization solutions. The authors in [15] proposed 2 novel continuous wavelet transformation (CWT)-based feature sets; an image-based CWT feature set from white gaussian noise augmented fingerprint RSSI signals and a numerical Power Spectral Density feature set (PSD) extracted from the CWT images (PSD-CWT). Results on both room-level and position-level indoor localization revealed that these feature sets perform better than conventional feature sets with the image-based CWT feature achieving higher accuracy results. Wang et al. [16] applied a Gaussian and a bootstrap filter to remove noise from RSSI signals. Results from a typical indoor environment and an anechoic chamber prove that the filtering algorithm reduces the localization error improving the accuracy. In [17] the issue of synchronization of wireless sensor nodes is being addressed. The authors propose a method that improves the mismatches among timestamps of inertial sensors.

Due to variations in RSSI measurements because of multipath fading and obstacles, resulting in errors in the position approximation, another common technique is to fuse RSSI data with data from other sensors. Depending on various types of sensors, there are many approaches for sensor fusion, offering a more robust indoor localization system. Nevertheless, there are two types of sensors for localization, namely relative position sensors, which compute the relative distance and/or orientation to a reference point, e.g., an accelerometer, and absolute position sensors, which compute the absolute position of the target, like sensors measuring RSSI. Most of the sensor fusion algorithms for localization are based on the fusion of RSSI data with inertial measurement unit (IMU) sensor data. Evennou and Marx [18] proposed a sensor fusion framework that uses a Kalman filter and a particle filter. The benefits of the Evennou and Marx architecture were evaluated and compared with pure WiFi localization systems and inertial navigation system (INS) positioning systems. In [19], Yoon et al. introduced an adaptive Kalman filter to combine BLE RSSI and tilt and yaw from IMU data. Malyavej et al. [20] built an indoor robot localization system, using an extended Kalman filter to combine position and velocity information from RSSI and IMU data.

In the same direction, Poulose et al. [21] combined WiFi RSSI data with IMU data for a robust indoor localization. Their model uses a WiFi fusion algorithm, using both RSSI-based trilateration and fingerprint results. The WiFi fusion algorithm results are then combined with results from a pedestrian dead reckoning (PDR) algorithm using the IMU sensor data. Their algorithm achieved a reasonably low localization error. In [22], the IMU data are processed by a sequential Monte-Carlo Kalman filter and integrated with WiFi RSSI positioning results. This method was found to rapidly drop the estimated error, compared to the extended Kalman filter. Kalman filter approaches have been developed to combine IMU and RSSI data and deal with scenarios with missing data [23]. RSSI and IMU-based location have been calculated independently in the work of [24]. The final estimation of localization is performed by integrating the two different location estimations.

Jeon et al. [25] suggest a method for indoor localization based on step detection from an accelerometer and magnetic sensor. They also used a barometer to estimate the level of the user’s floor by computing altitude changes. To mitigate cumulative errors of the proposed step detection method, the position estimation is corrected using BLE RSSI data. By using this method, they achieved higher accuracy compared to the simple step detection method.

The authors in [26] used the accelerometer and magnetometer sensors of a smartphone together with RSSI to improve the indoor localization accuracy. Their method is also based on step detection from the accelerometer data, and orientation detection from the magnetometer data. With these data, they attempted to improve the performance of the RSSI fingerprinting method for localization. Their results showed that using these sensors offers a reduction in the total off-track path length of a moving target from 16.353 m to 6.409 m. However, their algorithm’s performance in the case of a motionless target was poor. In [27] the authors proposed a scheme of RSSI and accelerometer fusion for indoor localization. A fingerprinting-based RSSI positioning and a weighted combination of the closest reference points’ estimations are used. For the accelerometer measurements, the authors used the Newton laws of movement considering constant acceleration between two consecutive time-steps. Finally, for the sensor fusion, the above calculations are computed taking into account the measurement incertitude, thus computing a position box from each sensor. The final position estimation is the center of the intersection of the RSSI and the accelerometer boxes. The authors tested their proposed algorithm using a simulation of a moving target with varying acceleration and they found promising results. Nevertheless, their method has not been tested in real-life data.

Data fusion methods have also been proposed for higher room-level localization performance. The ARIEL system [28] is based on dynamic room WiFi fingerprinting for a room-level localization estimation, using zone-based clustering and motion-based clustering to identify inter-zone correlation to distinguish different rooms. The motion-based clustering is performed using a low-frequency accelerometer, to differentiate between stationary and moving conditions of the user. The proposed system offered a high accuracy of 95% for room-level localization. The authors in [6] followed a different method for combining RSSI and accelerometer data. Their algorithm is based on the assumption that the location is also determined by the current activity of the user. They used feature extraction for the accelerometer data to improve simple fingerprinting-based RSSI positioning. Using different methods of combining RSSI and acceleration data they found that the best method was yet the simplest that is combing RSSI and features from raw acceleration data for localization. By implementing this method, they found an improved room-level localization estimation. In [29] the authors designed an automatic system able to perform region-level localization of firefighters in harsh environments. They proposed a system combining PDR and RSSI to achieve an average localization error of 5.6 m.

Kalman filtering techniques are the most chosen methods for the fusion of RSSI and inertial sensor readings when the actual distance needs to be predicted. Currently, some works are focusing on the fusion of RSSI and inertial measurements for room-level localization. Our work is contributing to the latter, by assessing the performance of fusion methods widely used in machine learning tasks and by using feature extraction for the elimination of noise in RSSI and accelerometer values. Previous research in the field of RSSI and IMU fusion for room-level localization has not offered such an analytic evaluation on multiple fusion methods.

## 3. Methods

In this section, we describe the algorithms applied for the fusion of the heterogeneous features, as well as the evaluation metrics employed to assess the performance of the classification. By the term fusion, we refer to the combination of data or results. Early fusion is implemented in the early stages of an analysis and is defined as the combination of raw data or extracted features. The most common form of early fusion is the concatenation, where the vectors of values of heterogeneous measurements are put together [30]. Late fusion refers to the combination of the results of the analysis, namely the classification results, whether in terms of predicted labels or in terms of predicted class probabilities. Fusion methods are also known as ensemble learning. In the current study, we chose six well-performing late fusion methods that are appropriate for the specific data. For the early fusion, we applied concatenation, where both the RSSI and the accelerometer features were bound by rows and inserted as input to the classification algorithms. In the following paragraphs, the applied late fusion methods are described.

The averaging fusion technique, is one of the simplest forms of late fusion [31] that uses the average of the predicted class probabilities of different classification algorithms and the class with the highest final probability is assigned to each case in the data. The final probability of each class is computed in Equation (1):

(1) $${\hat{P}}_{j}=\frac{\sum_{i=1}^{M}P_{i,j}}{\sum_{j=1}^{N}\sum_{i=1}^{M}P_{i,j}},$$

where $P_{i,j}$ is the probability of the *j* class of the *i*-th classifier, *M* is the total number of classifiers, and *N* the total number of classes.

Another class of late fusion algorithms are the ones that use weights. A very well-known algorithm is the accuracy weighted fusion algorithm [31], where the weights (Equation (2)) express the performance of each of the algorithms that will be combined, in terms of accuracy, an evaluation metric calculated by Equation (3). More specifically, a weighted sum is performed on the probabilities of each class and the class with the highest probability is chosen as the decision. Generally, in the weighted fusion techniques, the probability of each class is given by Equation (4).

(2) $$W_{i,j}=accuracy_{i}$$

(3) $$accuracy=\frac{TP+TN}{TP+FP+TN+FN}$$

where TP is true positives, the number of cases of a class correctly identified, TN is true negatives, the number of cases not belonging in a class that was correctly identified, FP is false positives, the number of cases not belonging to a class that was wrongly identified, and FN is false negatives, the number of cases belonging to a class that was wrongly identified.

(4) $${\hat{P}}_{j}=\sum_{i=1}^{M}P_{i,j}W_{i,j}$$

with $P_{i,j}$ being the probability of the *j* class of the *i*th classifier, and $W_{i,j}$ the weight of the *j* class of the *i*th classifier.

Another weighted fusion method applied in this work, is the Detection Rate weighted (DR weighted) fusion that was recently proposed by the authors of this paper. Instead of using the overall performance of each classifier to calculate the weights, the DR weighted fusion is based on the performance of the classifier in predicting each class. The detection rate (Equation (5)) is a strict evaluation metric that rates the classifier’s ability to detect true positive cases among the predictions. The weights are calculated based on the detection rate of each class, with the formula given in Equation (6). The weights are equal to the supplementary of detection rate to assist in the recognition of classes that are more difficult to be predicted. A weighted sum of the probabilities is calculated according to Equation (4) [32]. The final decision is the class with the highest final probability. The final probabilities are normalized so that the total sum of all probabilities for every class is one.

(5) DR=TP/(TP+TN+FP+FN)

(6) $$W_{i,j}=1-DR_{i,j},$$

where $DR_{i,j}$ is the detection rate of the *j* class and of the *i*-th classifier.

To optimize the selection of the weights for the classifiers, a genetic algorithm technique was followed. Genetic algorithms (GA) have been commonly used to solve optimization problems [33]. The main idea of genetic algorithms is that by creating an initial population of chromosomes, the algorithm mimics the natural selection in which the population is modified over time. A fitness function is used to determine which chromosomes of the population will survive. In each iteration, individuals that have survived are mutated to produce the children of the next generation. The steps followed in the genetic algorithm procedure are as follows:

(1) Initialization of the population of chromosomes.
(2) Selection of the part of the population that survive using the fitness function as a criterion.
(3) Creation of a new generation of chromosomes through a combination of genetic operators: crossover and mutation.
  - The crossover is a genetic operation used to combine two parents to create a new chromosome.
  - The mutation is a genetic operation used to maintain diversity from one generation to the next.
(4) Repetition of steps 2 and 3 until a termination condition is reached.

In our experiment, the weight of each classifier is represented in each gene of the chromosome. The fitness function chosen is the classification’s accuracy. The crossover rate, meaning the number of times a crossover occurs for chromosomes in one generation, is set to 0.8. The mutation rate is the rate that determines how many genes of each chromosome should be mutated in one generation. The mutation rate is set to 0.1. The feasible region is bounded by the constraint (0≤weight≤1). The total number of iterations for each classifier was 100.

A more complex late fusion method is stacking that involves training a classifier to combine the predictions of several other classifiers that are considered base learners [34]. In our experiments, Support Vector Machines (SVM) and Gradient Boosting Machine (GBM) classifiers were trained to combine the predictions of the same classifier trained on an accelerometer and RSSI data separately. Applying 10-times cross-validation, the out-of-fold probabilities of each classifier are obtained. These probabilities are used to train the SVM and the GBM classifier to perform the final prediction.

The different fusion methods were evaluated using the following metrics: accuracy, class-specific sensitivity and specificity. The accuracy is simply the rate of correct predictions over the total number of predictions (Equation (3)). When dealing with imbalanced data, accuracy is not always the optimal evaluation metric, since it performs poorly due to the most dominant class. This is known as the accuracy paradox. Other metric options for evaluation that are more class-specific, thus making them more relevant for imbalanced data, are sensitivity and specificity. Sensitivity measures the proportion of positives that are correctly identified (Equation (7)), while specificity measures the proportion of negatives that are correctly identified (Equation (8)), for each class [35].

(7) $$sensitivity=\frac{TP}{TP+FN}$$

(8) $$specificity=\frac{TN}{FP+TN}$$

High levels of sensitivity imply that the classifier can correctly identify a class when it occurs, and high levels of specificity mean that the classifier does not predict one class when it does not occur. Therefore, high levels of both sensitivity and specificity for each class mean that the classifier can distinguish the different classes and correctly predict them.

## 4. Results

### 4.1. Data

The dataset used in this research is presented in [11]. Using the EurValve Smart Home in a Box (SHiB) system [36], for each epoch, the provided data are the RSSI data from each gateway along with the receiving gateway, 5 acceleration samples, the timestamp, the sequence number of the packet sent from the SHiB wearable and the true room annotation that the user was in, as well as the associated activity (if available). The radio signal is broadcasted via BLE from the wrist-worn wearable. The accelerometer data are sampled at 20 Hz, thus at each epoch, 5 samples of acceleration are available. There are four gateways, each one placed in one of the following rooms: bedroom, kitchen, living room, and stairs.

The dataset consists of two parts; the first is the calibration part, which can be used for training classification algorithms and the second is the free-living or test part. For the first part, ten participants were involved in the four calibration sequences, to offer ground-truth calibration for the four rooms in the house. The participants were instructed to perform certain activities in each room; sitting in the living room, walking in the kitchen, standing on the stairs and lying in the bedroom.

The second part is the free-living dataset used as testing data. A participant was instructed to perform activities of a daily routine, like lying in bed and preparing a meal in the kitchen, wearing the wrist-worn wearable. The main difference between the calibration and the free-living data is the absence of the activity annotation from the second. Thus, the dataset is suitable for room-level localization, but no accurate activity recognition can be performed.

Before conducting the classification process, several features were extracted from the dataset. Feature extraction is a step often followed in inertial sensors’ measurements, like the accelerometer. However, in RSSI values we usually see the application of Kalman filters to eliminate noise. Since the goal in this work is to fuse accelerometers and RSSI values, we extracted the features proposed in [8]. First, we filter the RSSI and accelerometer values by applying a moving average for every 3 rows. Afterward, we extract the features using a moving window of 20 rows length with 50% overlap. In each of the following evaluation protocols, since the manipulation of the dataset was different, we resulted in different total numbers of variables and cases.

In the first evaluation protocol, we examined the room-level localization separately for each gateway. As it is expected, the RSSI values differ per gateway, as well as the true labels of the rooms in the range of each gateway. To match the timestamp of the RSSI incoming measurements, the five accelerometer samples accompanying each sequence number and RSSI value were treated as separate variables. From each one of these variables, the features of Table 1 were extracted, resulting in a total of 181 features per gateway dataset.

For the second evaluation protocol, by transposing the original data, the RSSI data was obtained for each gateway and each sequence number. The resulted transposed dataset consists of six columns, representing the sequence number, the RSSI data for each gateway (bedroom, kitchen, living room and stairs), and the labels of the true room. If any RSSI data for a gateway were missing, meaning that the gateway was not in range, its value was replaced with −120 dB. By applying a sliding window of 10 s with a 50% overlap, feature extraction was performed in the RSSI data. The features used for the classification can be seen in Table 1.

The acceleration data were extracted from the original data by only keeping each unique sequence number. For each unique sequence number, 5 acceleration samples were available in the dataset, since the acceleration sampling rate was 20 Hz. A rearrange of the accelerometer data was performed so that the final dataset comprises five columns; the sequence number, acceleration data along the three axes (one sample per row) and the true room label. The magnitude was computed for each sequence and the feature extraction was performed on the magnitude data. Using the same sliding window, we extracted the same features.

**Figure 1:**
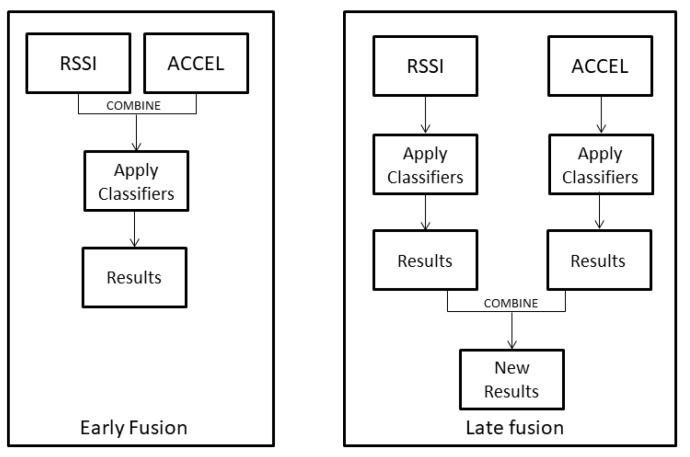
Application process in early (**left**) and late (**right**) fusion.

The classifiers chosen for the classification were Random Forest (RF) [37], k-Nearest Neighbors (kNN) [38], SVM [39] and Linear Discriminant Analysis (LDA) [40]. The classifiers were trained separately for RSSI and the acceleration features. We used concatenation for feature level fusion, and several decision level fusion schemes. For the decision fusion process, we used SVM and GBM stacking, averaging of the predicted class probabilities, accuracy weighted fusion, detection rate weighted fusion, and GA-weighted fusion. The flowchart in Figure 1 graphically depicts the differences between the application of early fusion and any late fusion method.

### 4.2. Experimental Results

In the first evaluation protocol, the results are presented separately for each gateway. The results of the living room gateway are presented in Table 2 and Table 3. From Table 2 it can be seen that the RSSI offers a much higher accuracy compared with the accelerometer sensor. Nevertheless, the late fusion techniques (GA-weighted fusion and GBM stacking) improve the accuracy of classification when compared with the RSSI-only accuracy, meaning that the accelerometer sensor can offer useful knowledge for the localization. This does not stand although for the SVM classifier where the very poor performance of the accelerometer influences the fusion results. Comparing every classifier in terms of maximum accuracy, the RF classifier with the late GA-weighted fusion method outperforms the other classifiers. Since we are dealing with imbalanced classes in our data, it is important to also compute the sensitivity and specificity for each class. We calculated the sensitivity and specificity of the RF classifier with all the different fusion methods since the RF classifier is the one performing the best in terms of accuracy.

**Figure 2:**
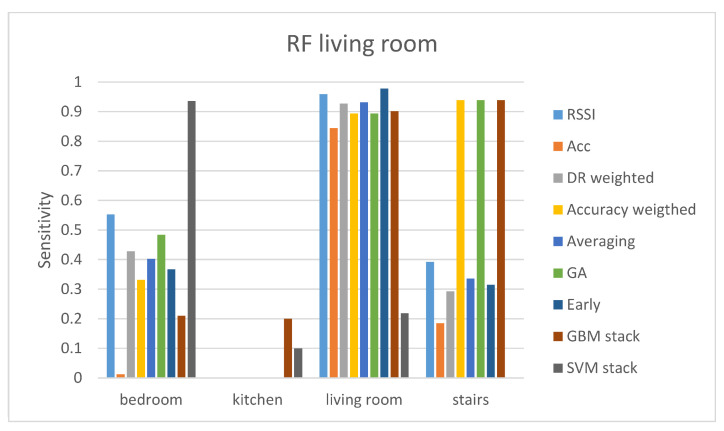
Sensitivity results of the RF classifier for the living room gateway.

**Figure 3:**
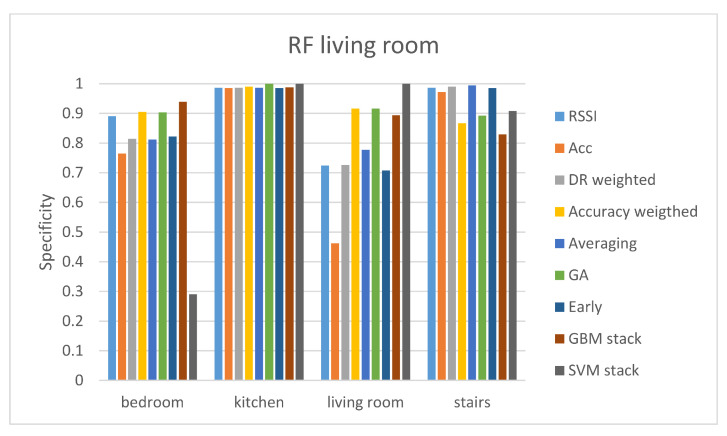
Specificity results of the RF classifier for the living room gateway.

Figure 2 and Figure 3 demonstrate the sensitivity and specificity results of the RF classifier with all the different fusion methods. It can be seen that even though the GA-weighted fusion achieves a relatively high total accuracy, it only performs well in two classes; the living room and stairs. The sensitivity and specificity results demonstrate that bedroom class does not have a high true predict rate (sensitivity<0.5) and that kitchen class is not predicted at all (sensitivity=0). Therefore, even though GA-weighted fusion has a total accuracy of 79.89%, it performs well only with two out of the four classes. It is worth mentioning that the two rooms that the living room gateway performs the best are those two that are the closest to the gateway. The kitchen room is far away from the living room gateway and is separated by walls while the bedroom is on another floor.

Table 4 and Table 5 present the results from the stairs gateway. In this gateway, the performance of the RSSI and accelerometer modalities is really poor. As a result, the performance of the different fusion techniques is also low. Again, it can be seen that fusion techniques (early fusion, GA-weighted fusion, and GBM stacking) improve the classification performance, but not by a great number. Out of all, the best accuracy is achieved by the LDA classifier using early level fusion. Again, we present the sensitivity and specificity values of all classes using the LDA classifier and all the fusion techniques.

**Figure 4:**
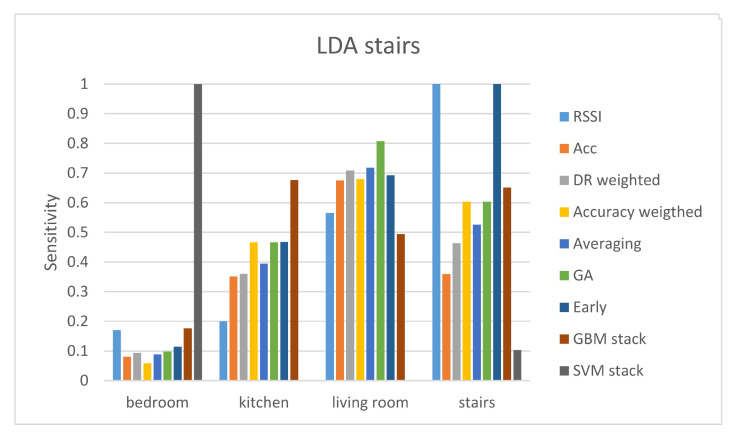
Sensitivity results of the LDA classifier for the stairs gateway.

**Figure 5:**
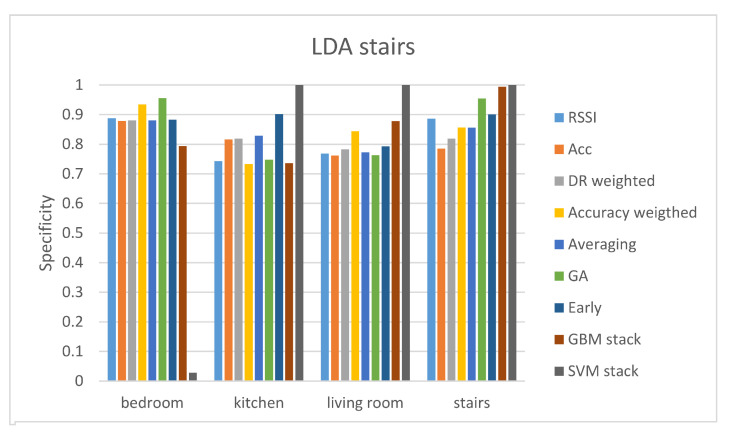
Specificity results of the LDA classifier for the stairs.

Results of the sensitivity and specificity of the LDA classifier are depicted in Figure 4 and Figure 5 respectively. Sensitivity results reveal that the only class with a high value of sensitivity is the stairs class. The predicted stairs class has a sensitivity value of 100% with both the RSSI and the early fusion. The RSSI values from the stairs gateway cannot offer a reliable prediction for any other class than stairs. Although early fusion improves the sensitivity and specificity values of the kitchen room as well. The really low performance in the prediction of three out of the four classes leads to relatively low accuracy of 61.98%. It is worth mentioning that the SVM stacking technique shows a perfect sensitivity score for the bedroom class, a very low score for the stairs class, and zero for the other two classes. Specificity results show that it has a perfect score for all classes but the bedroom. This means that the SVM stacking predicts almost every case as bedroom.

In the kitchen’s gateway, we observe good performance of both RSSI and accelerometer features (Table 6 and Table 7). The late fusion method of accuracy weighted fusion improves the classification results for all classifiers. Since the RSSI features perform quite well already, there is no room for significant improvement with the fusion of RSSI with the accelerometer values, except, probably, the case of the LDA classifier, that achieved accuracy around 80% for both modalities. In general, we can conclude that most of the fusion methods performed well in room-level localization. The highest value of accuracy is achieved from the RF classifier with both the accuracy weighted and GA-weighted fusion methods. The DR weighted late fusion performs worse than the individual sensors and the other fusion methods. This under-performance is probably caused by the unbalanced classes and by the fact that the method uses the supplementary of detection rate as weights, to boost the prediction of classes that are difficult to be predicted. However, this causes a bias in the prediction of classes with quite a few cases in the sample. It is worth mentioning that the accelerometer features fed in the SVM algorithm, predicted only the kitchen true room (accuracy= 0.9754, Table 6) and that the LDA algorithm using the RSSI features managed to predict even the one case of one living room as the true room.

In the bedroom gateway, the RSSI features individually, achieve very good accuracy levels in the room localization, while the accelerometer features perform poorly (Table 8). Subsequently, this affects the results of the fusion methods that do not achieve remarkable improvement of the individual performance of the RSSI features, as it can be seen in both Table 8 and Table 9. From this gateway, the room that was more easily recognized was the living room. In terms of accuracy, RSSI features, and GA-weighted fusion achieved the highest values, with the RF classifier with GA-weighted fusion method performing the best. In three out of the four classifiers used, the early fusion results are widely affected by the low performance of accelerometer performance. In conclusion, since one of the two modalities performs quite low, the fusion of the two modalities is not indicated.

The results of the proposed analysis of the second evaluation protocol are depicted in Table 10 and Table 11. It can be observed that when using information from all gateways at the same time the classification accuracy is improved. This was expected since, as seen in evaluation protocol 1, each of the four different gateways performs better classification for certain classes. Again, the accuracy results of the accelerometer are significantly lower than those of the RSSI. Fusion techniques (GA-weighted and concatenation) achieve improved accuracy compared to the RSSI ground-truth accuracy. In three of the four different classifiers (kNN, LDA, RF) the fusion method offering the best results in terms of accuracy is the GA-weighted fusion technique. The LDA classifier achieves the highest accuracy out of the rest.

**Figure 6:**
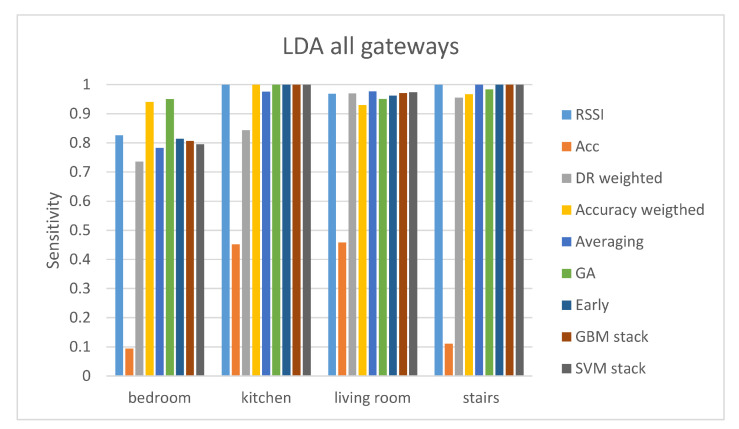
Sensitivity results of the LDA classifier for the evaluation protocol 2.

**Figure 7:**
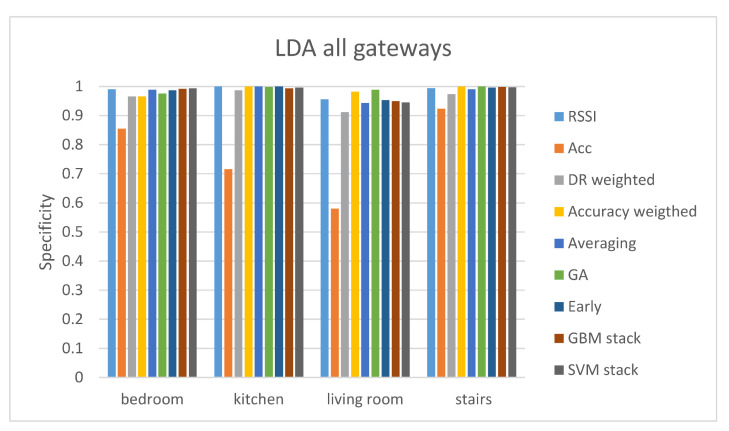
Specificity results of the LDA classifier for the evaluation protocol 2.

The sensitivity and specificity results are depicted in Figure 6 and Figure 7. These results show that the overall performance of all the fusion methods is good. Apart from the bedroom class, all the fusion methods offer sensitivity values higher than 90%, following the results of the RSSI predictions. The accuracy weighted and GA-weighted fusion are the only methods achieving a sensitivity value of over 90% for the bedroom class. The GA-weighted fusion method performs slightly better than the accuracy weighted algorithm in all the classes meaning that is the optimal method.

## 5. Discussion

RSSI values are known to have fluctuations and do not provide high accuracy in localization tasks. In this case, where the localization was performed at room-level and features were extracted from the RSSI measurements, the performance of RSSI was quite satisfactory. As our results demonstrated, there was no single classifier outperforming the rest in every scenario. Although RSSI features performed well in each gateway, the accelerometer features achieved good performance only in the kitchen gateway. Although only one activity was recorded per room, using the accelerometer, it did not distinguish the different rooms correctly on its own, except for results from the kitchen gateway. This might be an indication that the accelerometers assist in localization tasks that determine the exact distance between transmitter and receiver, where they are usually exploited to count steps and improve the distance predicted from the RSSI values.

The combination of RSSI and accelerometer data occurred in two different stages; feature level and decision level. The former aims to acquire information from different sources to improve classification performance. The latter assumes that the position of the user can also be determined by its activity. Information about activity obtained from the accelerometer and RSSI predictions about the position are combined to improve the performance of classification. In cases where the accelerometer’s individual performance was good, the fusion approaches achieve to improve the performance of the classification. The fusion methods applied, showed similar performance, except the DR weighted method that is probably not suitable for unbalanced classes. The two evaluation protocols did not produce significantly different results.

## 6. Conclusions

In this work, we assessed the performance of RSSI and accelerometer features in room-level localization. We performed individual analysis on each modality and applied early and late fusion to test their combined performance. In conclusion, we can argue that features extracted from the RSSI measurements along with filtering, improve the RSSI performance in room-level localization. Feature extraction from the RSSI values also enables the application of fusion algorithms that are used in classification tasks, instead of applying techniques like Kalman filtering to reduce signal noise. Although this particular application, resulted in poor performance of the accelerometer recordings, we firmly believe that with the use of other wearable devices the accelerometer could be of added value when combined with RSSI, for room localization. The fusion methods were naturally affected by the low performance of the one modality; however, some of them managed to increase the accuracy rate of the combined modalities, compared to the highest individual performance. For future work, we intend to continue the experimentation on the fusion of RSSI and accelerometer features for localization tasks, considering the use of different wearables, to test how and if they affect the results of fusion or the performance of the individual sensors and using different types of combination of the two modalities.

## Figures and Tables

**Table 1:** Features used for classification.

Features			
Mean	Standard deviation	25% quantile	Skewness
Median	Minimum	75% quantile	Kurtosis
Variance	Maximum	Interquartile range	

**Table 2:** Accuracy values for the living room gateway.

Classifier	RSSI	Acc	DR Weighted Fusion	Accuracy Weighted Fusion	Early Fusion	Averaging	GA Weighted
KNN	0.7506	0.4906	0.6622	0.7399	0.7466	0.6997	0.7614
LDA	0.6769	0.5402	0.5898	0.6662	0.6099	0.5416	0.7185
RF	0.7895	0.5201	0.7212	0.7668	0.7131	0.7439	0.7989
SVM	0.7386	0.1367	0.5268	0.7265	0.5389	0.5979	0.7212

**Table 3:** Accuracy values of stacking algorithms for the living room gateway.

Stacking Algorithms	KNN	LDA	RF	SVM
SVM	0.6769	0.6501	0.3539	0.4638
GBM	0.7319	0.7721	0.7493	0.5550

**Table 4:** Accuracy values for the stairs gateway.

Classifier	RSSI	Acc	DR Weighted Fusion	Accuracy Weighted Fusion	Early Fusion	Averaging	GA Weighted
KNN	0.4447	0.2304	0.3502	0.4539	0.5184	0.3871	0.4677
LDA	0.4954	0.4470	0.4908	0.5369	0.6198	0.5161	0.5922
RF	0.4516	0.4055	0.4839	0.5138	0.5276	0.4792	0.5553
SVM	0.5092	0.4217	0.3433	0.4147	0.5323	0.3433	0.4378

**Table 5:** Accuracy values of stacking algorithms for the stairs gateway.

Stacking Algorithms	KNN	LDA	RF	SVM
SVM	0.3249	0.1429	0.2419	0.3065
GBM	0.4470	0.5392	0.4355	0.5691

**Table 6:** Accuracy values for the kitchen gateway.

Classifier	RSSI	Acc	DR Weighted Fusion	Accuracy Weighted Fusion	Early Fusion	Averaging	GA Weighted
KNN	0.9526	0.9544	0.7807	0.9807	0.9561	0.9807	0.9860
LDA	0.8877	0.8018	0.7211	0.9298	0.5579	0.8439	0.9333
RF	0.9825	0.8105	0.6982	0.9912	0.9842	0.9842	0.9912
SVM	0.9754	0.9456	0.4825	0.9509	0.9561	0.9509	0.6175

**Table 7:** Accuracy values of stacking algorithms for the kitchen gateway.

Stacking Algorithms	KNN	LDA	RF	SVM
SVM	0.9649	0.9807	0.5782	0.7346
GBM	0.9543	0.8875	0.9859	0.9684

**Table 8:** Accuracy values for the bedroom gateway.

Classifier	RSSI	Acc	DR Weighted Fusion	Accuracy Weighted Fusion	Early Fusion	Averaging	GA Weighted
KNN	0.8372	0.4469	0.5448	0.8290	0.2414	0.7586	0.8524
LDA	0.8717	0.5228	0.5559	0.7917	0.7131	0.6221	0.8855
RF	0.8428	0.2869	0.4648	0.8303	0.4966	0.7393	0.8428
SVM	0.8593	0.2234	0.5214	0.8497	0.2414	0.6497	0.8386

**Table 9:** Accuracy values of stacking algorithms for the bedroom gateway.

Stacking Algorithms	KNN	LDA	RF	SVM
SVM	0.7793	0.8359	0.6455	0.6759
GBM	0.7945	0.8276	0.6386	0.6703

**Table 10:** Accuracy values for the evaluation protocol 2.

Classifier	RSSI	Acc	DR Weighted Fusion	Accuracy Weighted Fusion	Early Fusion	Averaging	GA Weighted
KNN	0.9390	0.3009	0.8573	0.9351	0.9364	0.8936	0.9429
LDA	0.9610	0.3307	0.8832	0.9598	0.9572	0.9481	0.9715
RF	0.9455	0.3929	0.7937	0.9351	0.9364	0.8716	0.9468
SVM	0.9416	0.3942	0.7886	0.9274	0.9572	0.8690	0.9429

**Table 11:** Accuracy values of stacking algorithms for the evaluation protocol 2.

Stacking Algorithms	KNN	LDA	RF	SVM
SVM	0.8872	0.9572	0.9183	0.9092
GBM	0.9339	0.9585	0.9429	0.9092

## Data Availability

The data used in this study are available in [11].

## References

[1] McConville R., Byrne D., Craddock I., Piechocki R., Pope J., Santos-Rodriguez R. Understanding the quality of calibrations for indoor localisation. Proceedings of the 2018 IEEE 4th World Forum on Internet of Things (WF-IoT).

[2] Hossain M.S. (2015). Cloud-supported cyber–physical localization framework for patients monitoring. IEEE Syst. J..

[3] Correa A., Barcelo M., Morell A., Vicario J.L. (2017). A review of pedestrian indoor positioning systems for mass market applications. Sensors.

[4] Ansari A.R., Saeed N., Haq M.I.U., Cho S. (2018). Accurate 3D localization method for public safety applications in vehicular ad-hoc networks. IEEE Access.

[5] Kyritsis A.I., Kostopoulos P., Deriaz M., Konstantas D. A BLE-based probabilistic room-level localization method. Proceedings of the 2016 International Conference on Localization and GNSS (ICL-GNSS).

[6] Kozlowski M., Byrne D., Santos-Rodriguez R., Piechocki R. Data Fusion for robust indoor localisation in digital health. Proceedings of the 2018 IEEE Wireless Communications and Networking Conference Workshops (WCNCW).

[7] Sadowski S., Spachos P. (2018). Rssi-based indoor localization with the internet of things. IEEE Access.

[8] McConville R., Archer G., Craddock I., Kozłowski M., Piechocki R., Pope J., Santos-Rodriguez R. (2021). Vesta: A digital health analytics platform for a smart home in a box. Future Gener. Comput. Syst..

[9] Tudor-Locke C., Barreira T.V., Schuna J.M. (2015). Comparison of step outputs for waist and wrist accelerometer attachment sites. Med. Sci. Sport. Exerc..

[10] Kozłowski M., Santos-Rodríguez R., Piechocki R. (2019). Sensor modalities and fusion for robust indoor localisation. EAI Endorsed Trans. Ambient Syst..

[11] McConville R., Byrne D., Craddock I., Piechocki R., Pope J., Santos-Rodriguez R. (2019). A dataset for room level indoor localization using a smart home in a box. Data Brief.

[12] Bahl P., Padmanabhan V.N. RADAR: An in-building RF-based user location and tracking system. Proceedings of the IEEE INFOCOM 2000, Conference on Computer Communications, Nineteenth Annual Joint Conference of the IEEE Computer and Communications Societies (Cat. No. 00CH37064).

[13] Husen M.N., Lee S. Indoor human localization with orientation using WiFi fingerprinting. Proceedings of the 8th International Conference on Ubiquitous Information Management and Communication.

[14] Poulose A., Han D.S. (2021). Hybrid Deep Learning Model Based Indoor Positioning Using Wi-Fi RSSI Heat Maps for Autonomous Applications. Electronics.

[15] Ssekidde P., Steven Eyobu O., Han D.S., Oyana T.J. (2021). Augmented CWT Features for Deep Learning-Based Indoor Localization Using WiFi RSSI Data. Appl. Sci..

[16] Wang J., Hwang J.G., Peng J., Park J., Park J.G. Gaussian Filtered RSSI-based Indoor Localization in WLAN using Bootstrap Filter. Proceedings of the 2021 International Conference on Electronics, Information, and Communication (ICEIC).

[17] Coviello G., Avitabile G. (2020). Multiple synchronized inertial measurement unit sensor boards platform for activity monitoring. IEEE Sens. J..

[18] Evennou F., Marx F. (2006). Advanced integration of WiFi and inertial navigation systems for indoor mobile positioning. EURASIP J. Adv. Signal Process..

[19] Yoon P.K., Zihajehzadeh S., Kang B.S., Park E.J. Adaptive Kalman filter for indoor localization using Bluetooth Low Energy and inertial measurement unit. Proceedings of the 2015 37th Annual International Conference of the IEEE Engineering in Medicine and Biology Society (EMBC).

[20] Malyavej V., Kumkeaw W., Aorpimai M. Indoor robot localization by RSSI/IMU sensor fusion. Proceedings of the 2013 10th International Conference on Electrical Engineering/Electronics, Computer, Telecommunications and Information Technology.

[21] Poulose A., Kim J., Han D.S. (2019). A sensor fusion framework for indoor localization using smartphone sensors and Wi-Fi RSSI measurements. Appl. Sci..

[22] Li W.W.L., Iltis R.A., Win M.Z. A smartphone localization algorithm using RSSI and inertial sensor measurement fusion. Proceedings of the 2013 IEEE Global Communications Conference (GLOBECOM).

[23] Xu Y., Shen T., Chen X.Y., Bu L.L., Feng N. (2019). Predictive adaptive Kalman filter and its application to INS/UWB-integrated human localization with missing UWB-based measurements. Int. J. Autom. Comput..

[24] Brigalda D., Tapus N. Indoor localization with correction and validation. Proceedings of the 2020 19th RoEduNet Conference: Networking in Education and Research (RoEduNet).

[25] Jeon J.S., Kong Y., Nam Y., Yim K. An indoor positioning system using bluetooth RSSI with an accelerometer and a barometer on a smartphone. Proceedings of the 2015 10th International Conference on Broadband and Wireless Computing, Communication and Applications (BWCCA).

[26] Grzechca D., Wróbel T., Bielecki P. Indoor localization of objects based on RSSI and MEMS sensors. Proceedings of the 2014 14th International symposium on communications and information technologies (ISCIT).

[27] Lv X., Mourad-Chehade F., Snoussi H. Fingerprinting-based localization using accelerometer information in wireless sensor networks. Proceedings of the 2013 IEEE Global Communications Conference (GLOBECOM).

[28] Jiang Y., Pan X., Li K., Lv Q., Dick R.P., Hannigan M., Shang L. Ariel: Automatic wi-fi based room fingerprinting for indoor localization. Proceedings of the 2012 ACM Conference on Ubiquitous Computing.

[29] Li J., Xie Z., Sun X., Tang J., Liu H., Stankovic J.A. An automatic and accurate localization system for firefighters. Proceedings of the 2018 IEEE/ACM Third International Conference on Internet-of-Things Design and Implementation (IoTDI).

[30] Gunes H., Piccardi M. Affect recognition from face and body: Early fusion vs. late fusion. Proceedings of the 2005 IEEE international conference on systems, man and cybernetics.

[31] Maghsoudi Y., Alimohammadi A., Zoej M.V., Mojaradi B. (2006). Weighted combination of multiple classifiers for the classification of hyperspectral images using a genetic algorithm. ISPRS Commission I Symposium” From Sensors to Imagery.

[32] Tsanousa A., Meditskos G., Vrochidis S., Kompatsiaris I. A weighted late fusion framework for recognizing human activity from wearable sensors. Proceedings of the 2019 10th International Conference on Information, Intelligence, Systems and Applications (IISA).

[33] Goldberg D.E., Deb K. (2000). Special issue on genetic algorithms. Comput. Methods Appl. Mech. Eng..

[34] Su X., Tong H., Ji P. (2014). Activity recognition with smartphone sensors. Tsinghua Sci. Technol..

[35] Yerushalmy J. (1947). Statistical problems in assessing methods of medical diagnosis, with special reference to X-ray techniques. Public Health Reports 1896–1970.

[36] Pope J., McConville R., Kozlowski M., Fafoutis X., Santos-Rodriguez R., Piechocki R.J., Craddock I. Sphere in a box: Practical and scalable eurvalve activity monitoring smart home kit. Proceedings of the 2017 IEEE 42nd Conference on Local Computer Networks Workshops (LCN Workshops).

[37] Ho T.K. Random decision forests. Proceedings of the 3rd international conference on document analysis and recognition.

[38] Hodges J.L. (1950). Discriminatory Analysis.

[39] Cortes C., Vapnik V. (1995). Support-vector networks. Mach. Learn..

[40] Ripley B.D. (2002). Modern Applied Statistics with S. Statistics and Computing.

